# Prevalence of Extended-Spectrum β-Lactamases in Multidrug-Resistant* Klebsiella pneumoniae* Isolates in Jordanian Hospitals

**DOI:** 10.1007/s44197-023-00096-2

**Published:** 2023-04-24

**Authors:** Suhaila A. Al-Sheboul, Ghina S. Al-Madi, Brent Brown, Wail A. Hayajneh

**Affiliations:** 1 Department of Medical Laboratory Sciences, Faculty of Applied Medical Sciences, Jordan University of Sciences and Technology (JUST), Irbid, Jordan; 2 Biochem123, London, NW7 4AU UK; 3 Department of Pediatrics and Neonatology, Faculty of Medicine and King Abdullah University Hospital, Jordan University of Science and Technology (JUST), Irbid, Jordan; 4 Children’s National Hospital, Saint Louis University, St. Joseph’s University Medical Center, Paterson, USA

**Keywords:** Extended-spectrum β-lactamases, Hematological malignancy, Jordan, *Klebsiella pneumoniae*, Multidrug resistance, Plasmid profile

## Abstract

The increase in the prevalence of infections caused by certain bacteria, such as *Klebsiella pneumonia* (*K. pneumoniae*), is a global health concern. Bacterial production of an enzyme called extended-spectrum beta-lactamase (ESBL) can generate resistance to antimicrobial therapeutics. Therefore, between 2012 and 2013, we investigated *K. pneumoniae* that produce ESBLs with the prevalence of individual genes including *bla*_SHV_, *bla*_CTX-M_, *bla*_TEM_, and *bla*_OXA_ isolated from clinical samples. A total of 99 variable diagnostic samples including blood from hematological malignancies (*n* = 14) or other clinical sources including sputum, pus, urine, and wound (*n* = 85) were analyzed. All samples' bacterial type was confirmed and their susceptibility to antimicrobial agents was established. Polymerase chain reaction (PCR) amplification was carried out to ascertain presence of specific genes that included *bla*_SHV_, *bla*_CTX-M_, *bla*_TEM_, and *bla*_OXA._ Plasmid DNA profiles were determined to assess significance between resistance to antimicrobial agents and plasmid number. It was found that among non-hematologic malignancy isolates, the highest rate of resistance was 87.9% to imipenem, with lowest rate being 2% to ampicillin. However, in hematologic malignancy isolates, the highest microbial resistance was 92.9% to ampicillin with the lowest rate of resistance at 28.6% to imipenem. Among collected isolates, 45% were ESBL-producers with 50% occurrence in hematologic malignancy individuals that were ESBL-producers. Within ESBL-producing isolates from hematologic malignancy individuals*, bla*_SHV_ was detected in 100%, *bla*_*CTX*-M_ in 85.7%, and *bla*_TEM_ and *bla*_OXA-1_ at 57.1% and 27.1%, respectively. In addition, *bla*_SHV,_ *bla*_CTX-M_, and *bla*_OXA_ were found in all non-hematological malignancy individuals with *bla*_TEM_ detected in 55.5% of samples. Our findings indicate that ESBLs expressing *bla*_SHV_ and *bla*_CTX-M_ genes are significantly prevalent in *K. pneumoniae* isolates from hematologic malignancy individuals. Plasmid analysis indicated plasmids in isolates collected from hematological malignancy individuals. Furthermore, there was a correlation between resistance to antimicrobial agents and plasmids within two groups analyzed. This study indicates an increase in incidence of *K. pneumoniae* infections displaying ESBL phenotypes in Jordan.

**Supplementary Information:**

The online version contains supplementary material available at 10.1007/s44197-023-00096-2.

## Introduction

### Overview

**Fig. 1:**
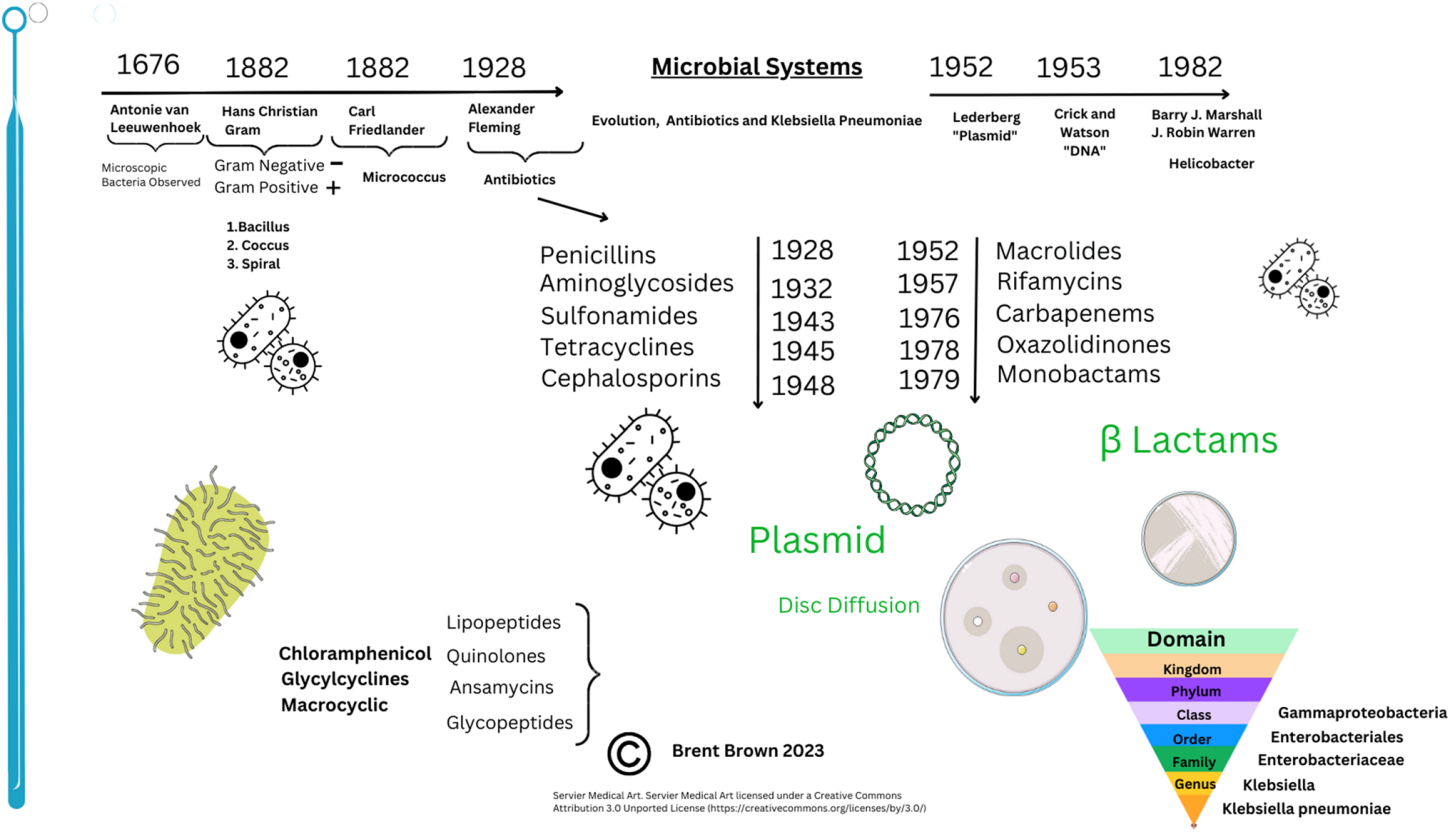
Antibiotics through history

*Klebsiella* is a Gram-negative, rod-shaped, encapsulated bacterium. It is a component of flora of the mouth, skin, and intestines initially discovered in 1882 by Friedlander as isolated from the lungs. *Klebsiella *spp. is classified as a member of the *Enterobacteriaceae* family and recognized as a cause of community and nosocomial acquired infections. *Enterobacteriaceae* can develop resistance to β-lactam antibiotics by different mechanisms; one of which is the plasmid-encoded ESBLs. *Klebsiella pneumoniae* that produce ESBLs are identified in hospitals worldwide [1]. A number of infections, including pneumonia, urinary tract infections (UTI), septicemia, but also wound infections can be caused by *Klebsiella *species (*Klebsiella* spp.) [1, 2]. However, treatment of infections caused by *Klebsiella* spp. is a therapeutic challenge for clinicians due to changes in incidence and prevalence of *Klebsiella* spp. that changes with resistance to antibiotic types alongside prior pathogenic exposure since initial discovery of antibiotics in 1928 by Alexander Fleming [3]. Subsequent characterization of antibiotics is shown below (see Fig. 1).

*Klebsiella* spp. affects immunocompromised hospitalized individuals including those with hematological malignancies [4, 5]. Subsequent infection can affect prognosis and cause varying morbidity and mortality in vulnerable individuals. As an opportunistic pathogen, *K. pneumoniae* is estimated to occur in between 3 and 8% of healthcare-associated bacterial infections (HCAI) and is indicated that in the nasopharynx, this occurs in 1–6% of individuals and also 5–38% of stool samples [6]. Data have similarly been reported in UK, Uganda, and recently Germany [7, 8]. *Klebsiella pneumoniae* is a key species of the genus and the most isolated *Klebsiella* spp. in human clinical samples [1, 9]. Another *Klebsiella* spp., *Klebsiella oxytoca*, is frequently isolated from human samples. Complications in individuals with hematological malignancies are quite common [10]. Hematologic malignancy individuals are immunocompromised due to the cellular effects of malignant cell cycles and administration of intensive immunosuppressive chemotherapy that affects both the lymphatic system, tissues, and cells [11]. These individuals are susceptible to infection by other pathogens that include fungi and viruses [12]. Other *Klebsiella* spp. are infrequently identified in clinical samples, such as *Klebsiella ozaenae*, *Klebsiella planticola*, *Klebsiella ornithinolytica*, *Klebsiella terrigena*, and *Klebsiella rhinoscleromatis* [13]. Different *Klebsiella* spp. show varying antimicrobial resistance (AMR) mechanisms.

### Brief History of ESBL-Producing *Klebsiella* Species

Initial reports of ESBL-producing *Klebsiella* spp. occurred around 1983 in Germany, with outbreaks reported worldwide [14]. *Klebsiella pneumoniae* was reported as the most common among bacterial species producing ESBLs [15]. In Jordan, a study was conducted among Jordanian ICU patients in 2000 that demonstrated *K. pneumoniae* isolates expressing an ESBL phenotype accounting for 70% of all isolates and this prevalence rate was found to be unusually high [16]. Individuals with hematologic malignancies therefore can be potentially hospitalized for longer as a result with increase the risk of morbidity and mortality [17–20]. A study in 2007 in Czech Republic identified the prevalence of *K. pneumoniae* in individuals with hematologic malignancies revealed that 11.4% of these bacterial infections could be caused by *K. pneumonia* [20, 21]. Subsequently, in 2010, reports appeared that *K. pneumoniae* caused UTIs in 10.2% of individuals with hematologic malignancies [20, 22]. On the other hand, a systematic review recently conducted by Nasser et al. highlighting the difference in antibiotic resistance patterns among *Escherichia coli* and ESKAPE pathogens (*Enterococcus faecium, Staphylococcus aureus, K. pneumoniae*, *Acinetobacter baumannii*, *Pseudomonas aeruginosa, and Enterobacter* spp.) in different countries in the Arabic region [23, 24]. These results highlight the difficulty in choosing antimicrobials for treatment to individuals.

### Antimicrobial Resistance and ESBLs

Antimicrobial resistance can commonly occur by production of ESBLs [22]. ESBLs are enzymes encoded mainly by genes located within plasmids and target the β-lactam ring. Some ESBL-encoding genes are located within transposons or insertion sequences that facilitate DNA transfer between bacterial species [17, 25]. Genes encoding ESBLs located on plasmids can transfer between bacterial strains, thus facilitating transfer [17]. In Gram-negative bacteria, including *K. pneumoniae*, broad-range enzymes emerged because of overuse of β-lactam antibiotics such as *bla* genes. The *bla* gene encodes β-lactamases that can be resistant to different antibiotics named β-lactams, apart from carbapenems and cephamycins that are comparatively lesser affected, and recent reports quantify this as between 0.13 and 22% in individuals receiving these [19, 20]. Historically, penicillin-derived beta-lactam (β-lactam) antibiotics were described as penams, carbapenams, oxapenams (clavams), penems, carbapenems, cephems, carbacephems, oxacephems, and monobactams. On the other hand, ESBL-producing isolates are more resistant to certain types of antibiotics like aztreonam, penicillin, and cephalosporin [26]. ESBL enzymes hydrolyze and deactivate a variety of β-lactam antibiotics that include types of penicillin, cephalosporine and monobactam, that are only one of a few inhibited by β-lactamase inhibitors, such as clavulanic acid, tazobactam, and sulbactam [26]. ESBLs arise mostly due to mutations in β-lactamases encoded by *bla*_SHV_, *bla*_TEM_, and *bla*_CTX-M_ genes [19, 20]. These occur by amino acid substitutions that change the active enzyme site [27]. Plasmid-mediated *bla*_TEM-1,_ *bla*_TEM-2_ alongside chromosomal-mediated *bla*_SHV-1,_* bla*_SHV-5_, and *bla*_SHV-12_ are the most common variants. Strains expressing *bla*_CTX-M_ type have emerged in many countries during the past decade and there are now common *bla*_non-TEM_ and *bla*_non-SHV_ ESBL types. Other ESBL genes producing enzymes, such as *bla*_OXA_, *bla*_VEB_, *bla*_PER,_ and *bla*_GES_, are less frequently encountered, although those derived from *bla*_OXA_ are considered less virulent [27, 28]. Recently, numerous variants of the original TEM, SHV, CTX-M type, and OXA lactamases have originated but not all of them are ESBL-producers and can occur in different bacteria like *Escherichia coli* (see Supplementary Materials). Currently there are 187 *bla* type defined by mixture of β-lactamases or cephalosporin categories [29]. Spread of *bla* (CTX-M-type) and *bla* (PER-2) β-lactamase genes in clinical isolates from Bolivian hospitals containing bla_CTX-M_ (*n* = 642) was examined in 2006. It was observed that *Pseudomonas aeruginosa* and *Acinetobacter* spp. produced ESBL from isolates (*n* = 106) contained novel genes in different bacterial species [30]. According to a recent study, there are 27 oxacillinases (chromosomally-mediated OXA-type β-lactamases) that are ESBLs and most of these oxacillinases are derived from *bla*_oxa-2_ and *bla*_Oxa-10_ [31]. Meanwhile, OXA-2 and OXA-10 were found in *Pseudomonas aeruginosa* and are recently known as narrow-spectrum β-lactamases [32]. The gene OXA-1 has been linked to false-ESBL phenotypes in *E. coli* isolates and resistance to combinations of β-lactamase inhibitors, together with the loss of porins [33].

### Classification in Multidrug Resistance

The European Centre for Disease Control (ECDC) confirms multidrug resistance (MDR) defined as “acquired non-susceptibility to at least 1 antimicrobial agent in ≥ 3 antimicrobial categories”. In addition, extensive-drug resistance (XDR) is utilized to describe “non-susceptibility to at least one agent in all but ≤ 2 antimicrobial categories” with pan-drug resistance (PDR) described as “non-susceptibility to all agents in all antimicrobial categories” [34, 35]. Currently, limited data are available regarding the prevalence of *K. pneumoniae* and sensitivity to antimicrobial agents in individuals with hematologic malignancies in Jordan. Therefore, here, we investigate the prevalence and epidemiology of *K. pneumoniae* among 99 individuals with both hematological and non-hematological malignancies including other clinical isolates with variable diagnosis to identify genes contributing to ESBLs in isolates using PCR with a majority of β-lactamase and non-β-lactamase antimicrobials [36]. This knowledge may assist in bacterial infection control.

## Materials and Methods

### Sample Collection

A total of 99 bacterial isolates were collected from microbiology laboratories of the following hospitals: King Abdullah University Hospital (KAUH, Irbid, Jordan), King Hussein Medical Center (KHMC), as well as Jordan University Hospital (JUH) (Amman, Jordan). Prior to testing, all isolates were stored in 15% glycerol-supplemented Luria–Bertani medium at − 80 °C (Thermo Fisher Scientific. MA, USA). Isolates were obtained from clinical specimens including sputum, pus, blood, and other clinical sources (see Supplemental Data S1). Out of 99 collected bacterial isolates, 14 were isolated from individuals diagnosed with hematological malignancies. Sample collection was not biased to gender or any age group. For complete information regarding anonymized patient age, gender, and diagnosis, see Supplementary Data. The study was carried out with consent from the institutional review board of ethics committee at Jordan University of Science and Technology (2013/64/9).

### Bacterial Identification

All isolates were identified at KAUH, KHMC, and JUH microbiology laboratories to genus level. Confirmation of genus identification and further identification to species level were performed using the Microgen STREP ID kit (Catalogue# MID-62, Microgen, UK).

### Antimicrobial Susceptibility Testing

Kirby–Bauer disk diffusion method was performed on Mueller–Hinton agar (MHA) (Oxoid Ltd. Basingstoke Hampshire, UK) plates to determine susceptibility of β-lactam and non-β-lactam antibiotics with results interpreted according to Clinical and Laboratory Standards Institute (CLSI) guidelines (see Supplementary Materials). For confirmation, the susceptibility of the isolated strains was tested against two initial antibiotics (ceftazaidime and ceftriaxone in combination with clavulanic acid), and then, 12 types of antimicrobial agents were tested (Table 1 and Supplementary Data). All antimicrobial disks used in antimicrobial susceptibility testing were obtained from Bioanalyze (Turkey). Antimicrobial agents used in ESBL screening and phenotypic confirmatory testing obtained from Mast Diagnostics (UK). The strains were recorded as sensitive, intermediate, or resistant based on CLSI guidelines (see Supplemental Data). For disk diffusion method–Mueller–Hinton agar, two control strains were used for quality assurance (QC): ATCC 700603, an ESBL-producing *K. pneumonia* isolate (positive control), and ATCC 25922, an *Escherichia coli* isolate (negative control). The QC strains for antimicrobial testing were recommended in Clinical and Laboratory Standards Institute [CLSI] guidelines (see Supplementary Data).

**Table 1:** ESBL's frequency among isolates

	ESBL positive	ESBL negative	Total number
Total isolates	45	54	99
Isolates from individuals of various diagnosis	38	47	85
Isolates from hematological malignancy individuals	7	7	14

### Identification of ESBL-Positive *K. pneumoniae* Isolates and Phenotypic Confirmatory Testing

Initial susceptibility screening of *K. pneumoniae* isolates to both ceftazidime (30 µg) and ceftriaxone (30 µg) was assessed by disk diffusion method for screening of ESBL production according to CLSI recommendation (Supplementary Data). Confirmatory testing required use of both cefotaxime and ceftazidime alone or in combination with clavulanic acid (see Supplemental Data). The double-disk diffusion method was used, and a 0.5 McFarland bacterial suspension spread over Mueller–Hinton agar (Oxoid, Hampshire, UK). A disk of ceftazidime (30 µg) and a disk of ceftazidime in combination with clavulanic acid (30/10 µg) were placed at 20 mm apart and incubated overnight at 37 °C with results were compared by the disk diffusion method. An increase in zone diameter in the presence of clavulanate significantly (≥ 5 mm) compared to the inhibition zone around ceftazidime disk was interpreted as confirmatory of ESBL production.

### Plasmid DNA Extraction and Profiling

Plasmids were extracted from 14 samples of hematologic malignancy individuals, including both ESBL-positive and ESBL-negative isolates. In addition, 17 random samples collected from non-hematologic malignant individuals were chosen to be extracted according to the type of the sample. Plasmid extraction was performed using Promega PureYieldTM Plasmid Miniprep System (Catalogue #A1223, USA) and according to the manufacturer’s instructions. After plasmid extraction, the concentration of plasmid from each isolate was measured using a NanoDrop™ 1000 Spectrophotometer (Thermofisher Scientific, Wilmington, *USA*). 1.5 µl DNA was needed to measure plasmid concentration at wavelength of 260 nm. An appropriate volume of 10 µl of the extracted plasmid DNA obtained from hematological malignancy patient isolates was loaded after mixing with loading dye (KAPA BIOSYSTEMS, USA). Plasmids were electrophoresed through 0.8% agarose gel under 100 V for 2 h. Plasmid bands were visualized with Ethidium bromide staining under UV transilluminator (Biometra, Germany). Sizes of the bands were compared with the lambda HindIII digest ladder (New England, BioLabs).

## Results

### Identification of *K. pneumoniae*

All isolates were identified to species level based on colony morphology, lactose fermentation on MacConkey agar, and results obtained by the Microgen STREP ID kit. All 99 collected isolates were confirmed as *K. pneumoniae* and of these 94 isolates were of known sample collection method.

### Antimicrobial Susceptibility Testing

**Fig. 2:**
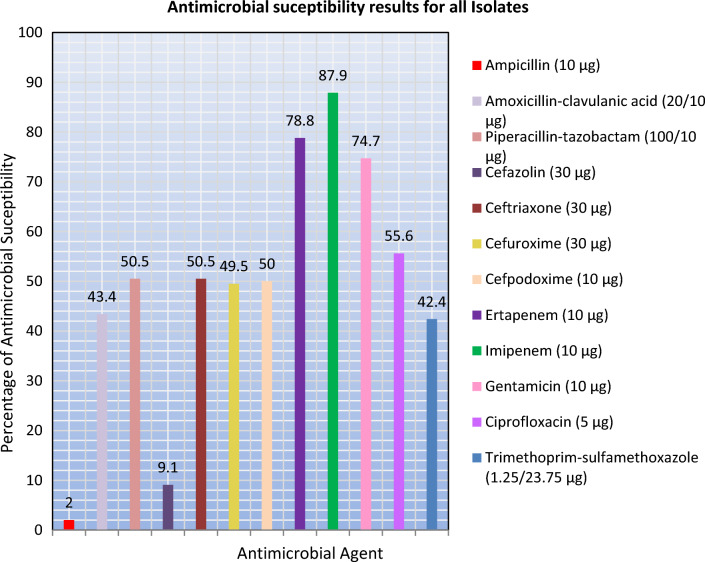
Antimicrobial susceptibility results for all isolates

**Fig. 3:**
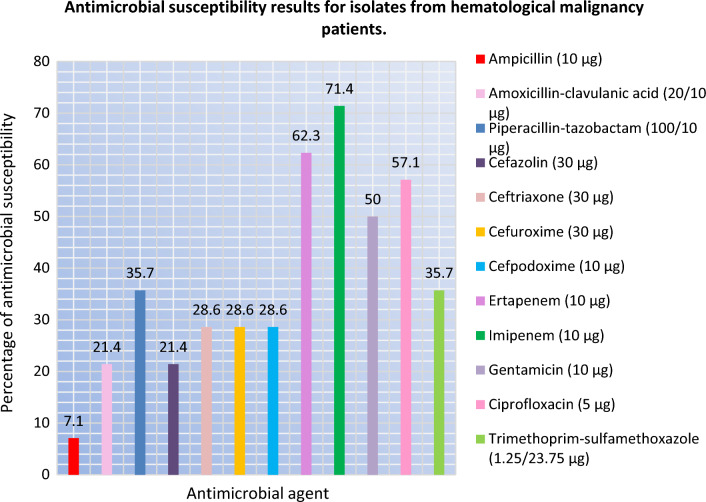
Antimicrobial susceptibility results for isolates from hematological malignancy patients

The antibiotic resistance pattern of *K. pneumoniae* isolates to different β-lactam and non-β-lactam antibiotics was variable. Overall percentage resistance of *K. pneumoniae* isolates against 12 selected antimicrobial agents recovered from clinical sources is compared (see Figs. 2 and 3 and Supplementary Data).

The majority of *K. pneumoniae* isolates therefore displayed resistance to different antibiotic drugs with variable resistance to each tested drug. The antibiogram for the 99 *K. pneumoniae* isolates in clinical samples are shown (Supplementary Data).

### ESBL Screening and Phenotypic Confirmatory Testing

Out of the 99 collected isolates, 45 isolates (45.5%) were ESBL-producers, while out of the 14 collected isolates from hematologic malignancy individuals, 7 isolates (50%) were ESBL-producers (Table 1).

### Detection of ESBL Genotypes by Polymerase Chain Reaction (PCR)

After PCR analysis of ESBL genotypes, all ESBL isolates that screened positive for *K. pneumoniae* were found to possess one or more ESBL genes tested (Table 2).

**Table 2:** Number of ESBL-positive or ESBL-negative isolates according to sample source and patient type

Sample source	Non-hematological individuals	ESBL positive	Hematological individuals	ESBL positive
Urine	59	23	4	2
Pus	9	5	1	1
Blood	8	4	2	1
Wound	5	3	0	0
Sputum	3	2	1	0
Tip of central line	1	1	1	1
Blood or urine	0	0	5	2
Total	85	38	14	7

Overall incidence of ESBL genotypes in screening positive isolates collected from hematological malignancy individuals showed that the frequency of ESBL-producers was: *bla*_TEM_ 57.1% (4/7), *bla*_SHV_ 100% (7/7), *bla*_OXA_ 27.1% (2/7), *bla*_*CTX-*M_ 85.7% (6/7) (Supplementary Data). In non-hematological malignancy (various diagnosis) individuals’ isolates, the frequency of ESBL-producers was as follows: *bla*_TEM_ 55.5%) % (5/9), *bla*_SHV_ 100% (9/9), *bla*_OXA_ 100% (9/9), and *bla*_*CTX-*M_ 100% (9/9).

### Plasmid Profiling

Detection of plasmids in hematological malignancies isolates using PCR indicates that isolates having high plasmid numbers (3–4 plasmids) in group B had higher resistance to various antimicrobials compared to group A isolates that have 1–2 plasmids with 57.1% in Group B compared to 42.9% in Group A (Table 3).

**Table 3:** Number of plasmids detected in isolates from hematologic malignancy individuals

Number of plasmids	Frequency of isolates from ESBL positive phenotype	Frequency of isolates from ESBL negative phenotype	Total number	Level of resistance profile
Group A: (1–2)	5 (35.7%)	1 (7.1%)	6 (42.9%)	Medium level (resistant to 1–6 antimicrobial agents)
Group B: (3–4)	2 (14.2%)	6 (42.9%)	8 (57.1%)	High level (resistant to 7–12 antimicrobial agents)

Plasmid profiling of 7 ESBL-positive and 7 ESBL-negative MDR-*K. pneumoniae* isolates detected plasmids in all the 14 isolates. The number of plasmids ranged from 1 to 4 plasmids among both ESBL-positive isolates and ESBL-negative isolates. The size of plasmids ranged from < 2 to 23 kb. Among ESBL-positive isolates and ESBL-negative isolates, there were two groups of plasmids; group A had medium level of resistance (1–6 antimicrobial agents) and group B showed high level of resistance (7–12 antimicrobial agents). A significant correlation was found between plasmid number and resistance to a number of antimicrobial agents. This correlation was established using Mann–Whitney test. The Mann–Whitney test established a significant relationship between plasmid number and level of antimicrobial resistance between group A and group B plasmids (*p* value of 0.029).

## Discussion

Members of the Enterobacteriaceae family are contributors to nosocomial and community-acquired infections and develop resistance to β-lactams via various mechanisms including ESBLs expressed by plasmids [37, 38]. *K. pneumoniae*, a member of this family, is the second most common cause of nosocomial infections in hospitals following *E. coli* [39].

In this study, 99 isolates of *K. pneumoniae* were collected from various types of clinical specimens from individuals with hematological (*n* = 14) and non-hematological malignancy (different sources with various diagnosis) (*n* = 85) from three major Jordanian hospitals. The frequency of *K. pneumoniae* infection among these individuals was assessed. Isolate resistance to antimicrobial agents and ESBL phenotype was identified and ESBL-encoding genes were characterized. It was determined that *K. pneumoniae* was the only species among *Klebsiella* spp. infecting all samples analyzed. Also, indicating that UTI is a common infection complication caused by *K. pneumoniae* in these individuals. Frequency of *K. pneumoniae* infections among hospitalized individuals was determined and resistance to antimicrobial agents was assessed specifically for ESBL-positive *K. pneumoniae* isolates. This evaluation provides significant data into antimicrobial resistance and *K. pneumoniae* phenotypes conducted in 2013 that may be a valuable insight into therapeutic treatment options.

Therefore, in other comparable studies in 2009, it was confirmed that 11.4% of *K. pneumoniae* infections with hematologic malignancies could be caused by *K. pneumoniae* [39]. Other studies conducted in Iran in 2014 (*n* = 300) showed that 38.1% of *K. pneumoniae* isolates had ESBL-producing phenotype [40, 41]. In contrast, a 1997 study (*n* = 97) conducted in Jordan indicated that 70% of *K. pneumoniae* isolates are with ESBL phenotype [16]. In Kuwait, prevalence of ESBL-producing *K. pneumoniae* was indicated at 12.2 vs. 11.4% using variable ESBL tests with UTI frequent in 2005 [42]. In contrast, in the United Arab Emirates in a comparison of *E. coli*, *K. pneumoniae*, and *Klebsiella oxytoca*. (*n* = 130), it was reported that 36% of *K. pneumoniae* were found to be ESBL-producers [42, 43]. In Saudi Arabia, depending on the geographical area the percentages varied between 12.2 and 55% [44]. In a global metadata study, the percentage of *K. pneumoniae* isolates was 43.5% with ESBL phenotype occurrence in this order: *bla*_SHV_ (24%), *bla*_CTX-M_ (28.1%), *bla*_TEM_ (25.2%), and *bla*_VEB_ (8.3%), respectively, between 2010 and 2018 [28]. However, the prevalence of ESBL phenotype in *K. pneumoniae and E. coli* varied among different European countries. In Europe, in a 2008 comparable report, *K. pneumoniae* was seen at 18.4% with ESBL-producers [45]. Whereas, ESBL-producing isolates in other countries is as follows: Latin America (45.4%), Europe (22.6%), Canada (4.9%), and USA (7.6%) [46]. The South American nations of Brazil, Venezuela, and Colombia were variable between 30 and 60% [29]. In India, the rates for *K. pneumonia, E. coli*, and *Klebsiella oxytoca* were 69.4%, 79.0%, and 100%, respectively [47].

Similarly, the prevalence rate of ESBL-producing *K. pneumoniae* in Ethiopia was around 17.1% [48]. However, in a larger study in Brazil (*n* = 1346) *K. pneumoniae* isolates that had an ESBL-producing phenotype was indicated at 43.7% to be predominantly *bla*_CTX_ and *bla*_*TEM*_ types [49]. While, Ivory Coast (*n* = 107) showed high prevalence of ESBLs’ producers among *K. pneumoniae* isolates with non-susceptibility indicated to the following antibiotics: sulfonamides (99%), quinolones (81%), and aminoglycosides (79%) [50].

However, in this study, antimicrobial susceptibility test (AST) among isolates having ESBL-producing phenotype demonstrated non-susceptibility of 98% to ampicillin, 90.9% to cefazolin, 57.6% to trimethoprim–sulfamethoxazole, 56.6% to amoxicillin–clavulanic acid, and 50.5% to cefuroxime, while 87.88% were susceptible to imipenem and 78.8% to ertapenem. We noticed that in comparison to the ESBL-non producers, the ESBL-producers appear resistant to β-lactams. The overall resistance rates of *K. pneumoniae* isolates to different classes of β-lactam antibiotics under study were very high for most antibiotics tested, except for carbapenems indicating 62.3% sensitivity to ertapenem and 71.4% to imipenem. On the other hand, a systematic review conducted by Nasser et al. highlighting the difference in antibiotic resistance patterns among *E. coli* and ESKAPE pathogens (*Enterococcus faecium, Staphylococcus aureus, K. pneumoniae, Acinetobacter baumannii, Pseudomonas aeruginosa, and Enterobacter* spp.) in different countries in the Arabic region [23]. These results highlight the difficulty in choosing antimicrobials for treatment. This may be because the present study was conducted on isolates collected between 2012 and 2013 period.

In our investigation, *K. pneumoniae* isolates had antibiotic resistance that was comparable to studies done in India, Romania, and other countries [51, 52]. Conventional PCR for the detection of genes encoding ESBLs showed that the dominant ESBL type in *K. pneumoniae* among individuals with hematological malignancy was SHV (100%), which is consistent with the other results. The *bla*_CTX-M_ type has a prevalence rate of 85.7% which is the most common *bla*_non-TEM_, *bla*_non-SHV_ type. Also *bla*_TEM_ and *bla*_OXA_ β-lactamase types had prevalence rates of 57.1% and 27.1% among individuals with hematologic malignancies. Among non-hematologic malignancy individuals, similar results were obtained, with *bla*_SHV_, *bla*_OXA_, and *bla*_CTX-M_ being dominant. In contrast, an Iranian study reported prevalence rates of 69.6% and 32.1% for *bla*_SHV_ and *bla*_TEM_ genes, respectively, among ESBL-producing *K. pneumoniae* [28]. In contrast, a study in Iraq showed the following ratios: 64.7% *bla*_TEM_, 35.2% *bla*_SHV_, and 41.1% *bla*_CTX-M_ genes existed in their isolates of *K. pneumoniae* [28].

Consequently, we found that four non-ESBL-generating isolates have ESBL-encoding genes. This could be due to truncation of ESBL-encoding genes, resulting in no expression of the genes. Additionally, there have been reports of other ESBL phenotypes with resistance, such as *bla*_TEM_ and *bla*_SHV_ β-lactamases with decreased affinities for β-lactamase inhibitors, AmpC-type enzyme production (both chromosomal and plasmid-mediated), as well as more complex ESBL phenotypes [53]. In the present study, seven ESBL-positive *K. pneumoniae* isolates, and seven ESBL-negative *K. pneumoniae* isolates from hematologic malignancies were subjected to plasmid profiling. One-to-four plasmids were present in ESBL-positive and ESBL-negative isolates. Plasmids ranged in size from 2 to 23 kb). There were two plasmid groups among the ESBL-positive and ESBL-negative isolates: Group A contained one to two plasmids (resistant to 1–6 antimicrobial drugs), whereas Group B had three-to-four plasmids and showed a high level of resistance (resistant to 7–12 antimicrobial agents). Resistance to several antimicrobial drugs and plasmid growth were significantly correlated.

In addition, we have investigated the presence of ESBL genes in non-ESBL-producers to evaluate the reliability of ESBL screening and phenotypic confirmatory testing. It was noted that four non-ESBL-producing isolates had ESBL-encoding genes, and this can be explained by the higher specificity and sensitivity of molecular methods compared to phenotypic screening as previous studies have suggested. Furthermore, increasing reports of more complex ESBL phenotypes that include additional mechanisms of resistance, such as AmpC-type enzyme production (both chromosomal and plasmid- mediated), TEM, and SHV β-lactamases with reduced affinities for β-lactamase inhibitors have been shown [53].

## Limitations

Low grant funding and insufficient sample size influenced study outcome. Additionally, the variations in results in our study compared to those found in the previous studies could be due to different testing procedures, hospital environments, and frequent use of different antimicrobials. Then again, resistance transfer experiments were not conducted; therefore, it is difficult to associate specific plasmids with β-lactam resistant phenotypes. Future studies is needed to address the plasmid types and the location of β-lactam encoding genes on the particular plasmids when testing for genes that has evolved since our 2013 study to uncover other relevant *K. pneumoniae* genes that produce ESBL. These genes include *bla*_OXA-48_, *bla*_CTX-M-15_, *bla*_KPC-2_, *bla*_OXA-9_, *bla*_SHV-11_, *bla*_SHV-5_, *bla*_CTX-M-3_, *bla*_CTX-M-14_, *bla*_VIM-1_, and the plasmid-encoded quinolone resistance (PMQR) gene [61]. Further research is underway to identify carbapenem genes (*bla*_*OXA-48*_*, bla*_*VIM*_*, bla*_*IMP*_*, bla*_KPC_*, bla*_NDM_, *bla*_KPC_) among immunocompromised population.

## Conclusion

Our study showed an increase in prevalence of MDR among *K. pneumoniae* individuals in Jordan especially those having ESBL phenotypes with hematologic malignancy. According to these results, further research and interventions should and can be done to limit and control the high frequency of ESBL-positive *K. pneumoniae*. Furthermore, the study shows the necessity for continuous surveillance and control of antibiotic resistance by the appropriate use of antibiotics.

## Supplementary Information

Below is the link to the electronic supplementary material.

Supplementary file1 (DOCX 127 KB)

## Data Availability

The following supporting information is included as a Supplementary Data in submission and is accessible. For further details on CLSI guidelines (2011) Wayne, P.A. Clinical and Laboratory Standards Institute. Performance standards for antimicrobial susceptibility testing. Updated version available March 2023 M100Ed32 | Performance Standards for Antimicrobial Susceptibility Testing, 32nd Edition (clsi.org). *Klebsiella* species bacteraemia: monthly data by location of onset—GOV.UK (https://www.gov.uk).

## Abbreviations

ESBLs: Extended-spectrum beta-lactamases
*K. pneumoniae*: *Klebsiella pneumoniae*
*E. coli*: *Escherichia coli*
MDR: Multidrug resistance
AMR: Antimicrobial resistance
HCAIs: Healthcare-associated infections
ECDC: The European Centre for Disease Control
XDR: Extensive-drug resistance
PDR: Pan-drug resistance
CLSI: Clinical Laboratory Standards Institute
JUST: Jordan University of Science and Technology
KAUH: King Abdullah University Hospital
KHMC: King Hussein Medical Center
JUH: Jordan University Hospital
