# Correction: Prevalence of Extended-Spectrum β-Lactamases in Multidrug-Resistant *Klebsiella pneumoniae* Isolates in Jordanian Hospitals

**DOI:** 10.1007/s44197-023-00120-5

**Published:** 2023-05-23

**Authors:** Suhaila A. Al-Sheboul, Ghina S. Al-Madi, Brent Brown, Wail A. Hayajneh

**Affiliations:** 1 Department of Medical Laboratory Sciences, Faculty of Applied Medical Sciences, Jordan University of Sciences and Technology (JUST), Irbid, Jordan; 2 Biochem123, London, NW7 4AU UK; 3 Department of Pediatrics and Neonatology, Faculty of Medicine and King Abdullah University Hospital, Jordan University of Science and Technology (JUST), Irbid, Jordan; 4 Children’s National Hospital, Saint Louis University, St. Joseph’s University Medical Center, Paterson, USA

**Correction: Journal of Epidemiology and Global Health** 10.1007/s44197-023-00096-2

Corrections were made to the abstract, section ‘4 Discussion’ and Fig. 1 of this article.

The second sentence of the abstract was incorrectly given as ‘Bacterial enzyme production of called extended-spectrum beta-lactamase (ESBL) can generate resistance to antimicrobial therapeutics.’ and should have read ‘Bacterial production of an enzyme called extended-spectrum beta-lactamase (ESBL) can generate resistance to antimicrobial therapeutics.’

The sixth sentence of the abstract was incorrectly given as ‘PCR amplification was carried out to ascertain presence of specific genes that included *bla*_SHV_, *bla*_CTX-M_, *bla*_TEM_, and *bla*_OXA_.’ and should have read ‘Polymerase chain reaction (PCR) amplification was carried out to ascertain presence of specific genes that included *bla*_SHV_, *bla*_CTX-M_, *bla*_TEM_, and *bla*_OXA_.’

The tenth sentence of the abstract was incorrectly given as ‘Of collected isolates, 45% were ESBL-producers with 50% occurrence in hematologic malignancy individuals that were ESBL-producers.’ and should have read ‘Among collected isolates, 45% were ESBL-producers with 50% occurrence in hematologic malignancy individuals that were ESBL-producers.’

The last sentence of the abstract was incorrectly given as ‘This study indicates an increase in prevalence of *K. pneumoniae* infections displaying ESBL phenotypes in Jordan.’ and should have read ‘This study indicates an increase in incidence of *K. pneumoniae* infections displaying ESBL phenotypes in Jordan.’

Reference [53] was added to the last sentence of section ‘4 Discussion’ which now reads: ‘Furthermore, increasing reports of more complex ESBL phenotypes that include additional mechanisms of resistance, such as AmpC-type enzyme production (both chromosomal and plasmid- mediated), TEM, and SHV β-lactamases with reduced affinities for β-lactamase inhibitors have been shown [53].’

Additionally, a wrong figure appeared as Fig. 1; the Fig. 1 should have appeared as shown below.

**Fig. 1:**
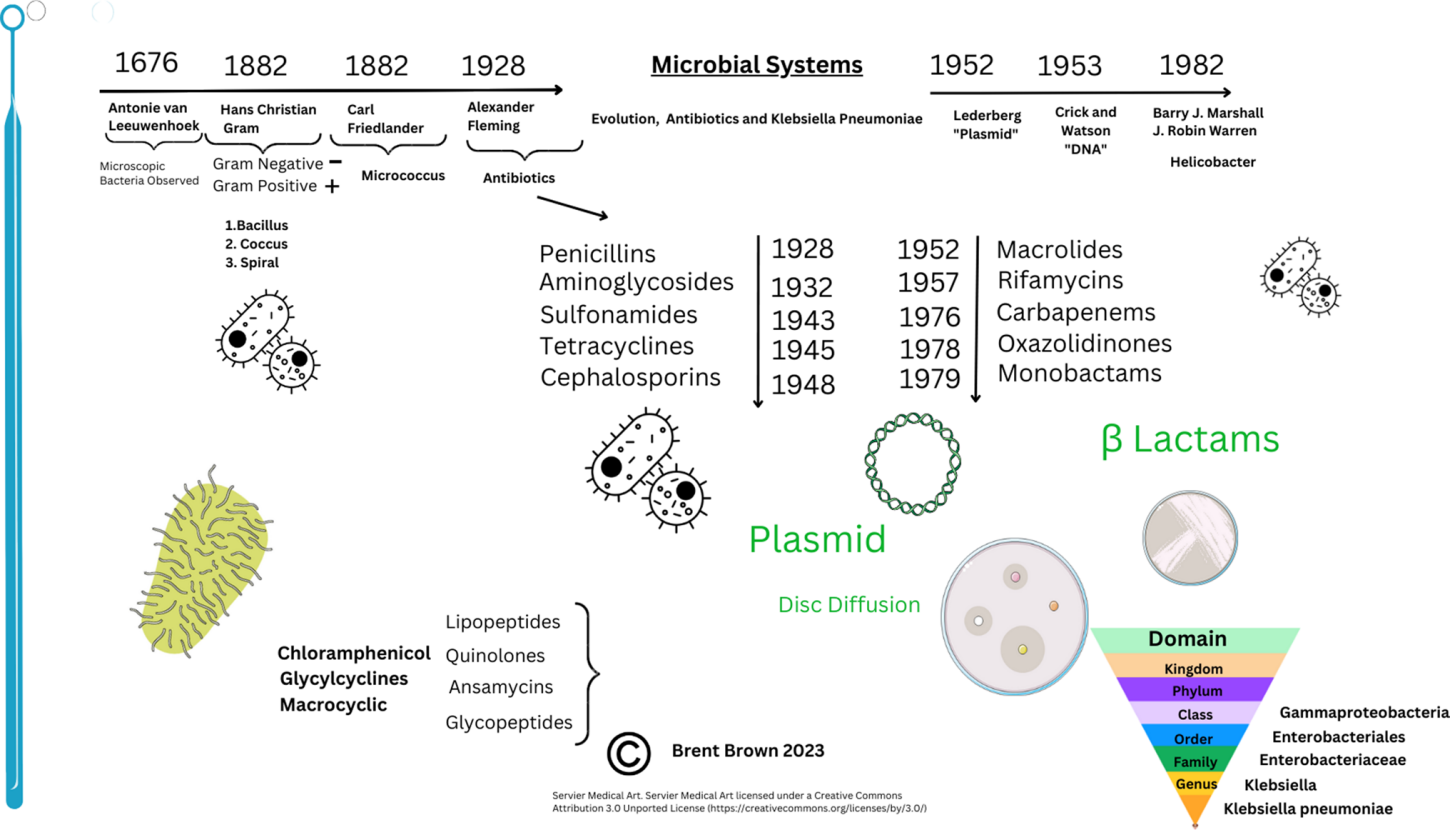
Antibiotics through history

The original article has been corrected.

