# Supplementary material for: Prevalence of Extended-Spectrum β-Lactamases in Multidrug-Resistant Klebsiella pneumoniae Isolates in Jordanian Hospitals
Source: J Epidemiol Glob Health. 2023 Apr 24;13(2):180–90. doi: 10.1007/s44197-023-00096-2 (PMC10272028; doi:10.1007/s44197-023-00096-2)
Supplement: Supplementary file 1 — Supplementary file1 (DOCX 127 KB) [file 44197_2023_96_MOESM1_ESM.docx]

Supplementary Data

Prevalence of Extended-Spectrum Beta-Lactamases In Multidrug-Resistant *Klebsiella Pneumoniae* Isolates in Jordanian Hospitals

Suhaila A. Al-Sheboul ^1,^*, Ghina S. Al-Madi ^1^, Brent Brown ^2^, Wail A. Hayajneh ^3,4^

^1^ Jordan University of Sciences and Technology (JUST), Department of Medical Laboratory Sciences, Faculty of Applied Medical Sciences. Irbid 22110, Jordan. sashboul@just.edu.jo; gmadi@hct.ac.ae

^2^ Director, Biochem123, London; NW7 4AU UK. info@biochem123.org, ORCiD-ID: orcid.org/0000-0001-5238-6943

^3^ Jordan University of Science and Technology (JUST), Department of Pediatrics and Neonatology, Faculty of Medicine and King Abdullah University Hospital. Irbid 22110, Jordan. wailh@just.edu.jo, ORCiD-ID: orcid.org/ 0000-0003-1816-9018)

^4^ Children's National Hospital, Saint Louis University, St. Joseph’s University Medical Center, USA. wailh@just.edu.jo

***** Correspondence: sashboul@just.edu.jo, ORCiD-ID: orcid.org/0000-0001-9001-3232

**Supplementary Data S1.** Grouping of samples collected from patients with variable diagnosis including hematological malignancies

| **Type of sample** | **Total number of samples** |
| --- | --- |
| Urine | 63 (63.64 %) |
| Pus | 10 (10.1%) |
| Blood | 10 (10.1%) |
| Wound | 5 (5%) |
| Sputum | 4 (4%) |
| Tip of central line | 2 (2%) |
| Unknown source | 5 (5%) |
| Total number | 99 samples |

70

60

50

40

30

20

10

0

Urineeeee

Pus

Blood

Wound Sputum Tip of central Unknown

line source

**Type of sample ofsample samplesample**

Supplementary Data S2. Patient’s gender, age, diagnosis and type of sample collected.

|  | **GGender** | **Age** | **Sample Type** | **Diagnosis** | **ESBL** |
| --- | --- | --- | --- | --- | --- |
| 1 | F | 56 Y | TCL | LLA | Positive |
| 2 | M | 36 Y | Pus | Nasal septal deviation with anosmia and nasal  crusting | Negative |
| 3 | M | 71 Y | Urine | UTI | Positive |
| 4 | M | 81 Y | Urine | UTI | Positive |
| 5 | F | 20 Y | Pus | Hodgkin disease | Positive |
| 6 | M | 74 Y | Urine | UTI | Negative |
| 7 | F | 50 Y | Pus | Peritoneal adhesions | Positive |
| 8 | F | 12 Y | Urine | UTI | Negative |
| 9 | F | 37 Y | Urine | UTI | Negative |
| 10 | F | 29 Y | Urine | UTI | Positive |
| 11 | F | 69 Y | Pus | Ovarian cancer (metastasis to liver and lungs) | Negative |
| 12 | M | 5 Y | Pus | Epidermolysis bullosa with  infected back ulcer | Negative |
| 13 | F | 31 Y | Urine | UTI | Negative |
| 14 | F | 48 Y | Pus | Aspiration pneumonia | Positive |
| 15 | F | 24 Y | Urine | UTI | Negative |
| 16 | M | 43 Y | Urine | UTI | Negative |
| 17 | F | 61 Y | Urine | UTI | Negative |
| 18 | F | 40 Y | Urine | UTI | Negative |
| 19 | M | 28 Y | Urine | UTI | Positive |
| 20 | M | 40 Y | Urine | UTI | Negative |
| 21 | F | 67 Y | Blood | Sepsis, Primary peritoneal  cancer | Positive |
| 22 | F | 7 Y | Urine | UTI | Negative |
| 23 | F | 45 Y | Urine | UTI | Positive |
| 24 | M | 41 Y | Urine | UTI | Positive |
| 25 | M | 69 Y | Blood | Sepsis | Positive |
| 26 | F | 20 Y | Sputum | Multiple trauma due to blast surgery | Positive |
| 27 | F | 15 Y | Urine | ALL , UTI | Negative |
| 28 | F | 23 Y | Urine | UTI | Negative |
| 29 | F | 42 Y | Urine | UTI | Negative |
| 30 | M | 81 Y | Urine | UTI | Positive |
| 31 | F | 47 Y | Urine | UTI | Negative |
| 32 | F | 45 Y | Urine | UTI | Positive |
| 33 | F | 11 Y | Urine | UTI | Positive |
| 34 | M | 63 Y | Wound  swab | Multiple lower limb ulcer,  Diabetes mellitus | Positive |
| 35 | F | 26 Y | Urine | UTI | Positive |
| 36 | M | 16 Y | Urine | UTI | Positive |
| 37 | F | 25 Y | Urine | UTI | Negative |
| 38 | F | 67 Y | Urine | UTI | Positive |
| 39 | M | 77 Y | Sputum | ALL | Negative |
| 40 | F | 50 Y | Urine | UTI | Negative |
| 41 | F | 46 Y | Urine | UTI | Positive |
| 42 | F | 45 Y | Wound  swab | Fall with acute infection | Positive |
| 43 | M | 47 Y | Wound swab | RTI | Negative |
| 44 | F | 26 Y | Urine | UTI | Negative |
| 45 | F | 26 Y | Urine | UTI | Negative |
| 46 | F | 63 Y | Urine | UTI | Positive |
| 47 | M | 51 Y | Urine | UTI | Negative |
| 48 | F | 6 Y | Blood | Sepsis, patient burn | Positive |
| 49 | F | 91 Y | Pus | Abdominal pain, gall bladder  Obstruction | Positive |
| 50 | F | 9 Month | Pus | Premature baby 29 week  presented with vomiting | Positive |
| 51 | M | 41 Y | Urine | UTI | Positive |
| 52 | F | 26 Y | Urine | UTI | Negative |
| 53 | M | 28 Days | Blood | Sepsis | Positive |
| 54 | F | 9 Y | Urine | UTI | Negative |
| 55 | F | 86 Y | Urine | UTI | Negative |
| 56 | M | 42 Y | Urine | UTI | Positive |
| 57 | M | 60 Y | Pus | Cholangitis | Negative |
| 58 | F | 77 Y | Urine | UTI | Negative |
| 59 | M | 86 Y | Urine | UTI, Prostate cancer | Negative |
| 60 | M | 18 Y | Urine | UTI | Positive |
| 61 | F | 47 Y | TCL | Stomach cancer | Positive |
| 62 | F | 19 Y | Urine | UTI | Negative |
| 63 | F | 3 month | Blood | Sepsis | Negative |
| 64 | M | 26 Y | Urine | UTI | Negative |
| 65 | F | One month | Blood | Sepsis | Negative |
| 66 | F | 26 Y | Urine | UTI | Positive |
| 67 | F | 60 Y | Sputum | Right sided pulmonary  embolism | Positive |
| 68 | F | 43 Y | Urine | UTI | Negative |
| 69 | F | 52 Y | Wound  swab | Hydatid cyst | Positive |
| 70 | F | 20 Y | Urine | UTI | Negative |
| 71 | M | 60 Y | Urine | UTI | Negative |
| 72 | M | One  Month | Wound  swab | Premature baby  Wound infection | Negative |
| 73. | M | 74 Y | Urine | UTI | Positive |
| 74 | M | 58 Y | doolB | Sepsis | Negative |
| 75 | F | 57 Y | Urine | UTI | Negative |
| 76 | F | 52 Y | Urine | UTI | Negative |
| 77 | M | 46 Y | Sputum | Bladder cancer | Negative |
| 78 | M | 69 Y | Urine | UTI | Positive |
| 79 | F | 23 Y | Urine | UTI | Negative |
| 80 | M | 58 Y | Urine | CLL,UTI | Positive |
| 81 | M | 73 Y | Urine | Prostate cancer, UTI | Negative |
| 82 | F | 50 Y | Pus | Diabetic foot | Positive |
| 83 | F | 27 Y | Urine | UTI | Positive |
| 84 | F | 32 Y | Urine | UTI | Positive |
| 85 | M | 64 Y | Urine | UTI,  Neutropenic fever, cholangiocarcinoma | Positive |
| 86 | F | 32 Y | Urine | UTI | Negative |
| 87 | F | 10 Y | Urine | UTI | Negative |
| 88 | F | 63 Y | Urine | UTI | Negative |
| 89 | F | Unknown | Urine | CML,UTI | Negative |
| 90 | M | Unknown | Urine | AML,UTI | Positive |
| 91 | M | 24 Y | Unknown | CML | Negative |
| 92 | F | 30 Y | Unknown | CML | Positive |
| 93 | M | 45 Y | Unknown | CLL | Positive |
| 94 | F | 44 Y | Blood | AML | Negative |
| 95 | F | 50 Y | Blood | AML | Positive |
| 96 | F | 41 Y | Blood | Squamous cell carcinoma-  tongue | Negative |
| 97 | F | 44 Y | Urine | UTI | Positive |
| 98 | F | 23 Y | Unknown | ALL | Negative |
| 99 | M | 55 Y | Unknown | CLL | Negative |

**Supplementary Data S3**. Antibiogram for all isolates

| **Isolates number** | **AM** | **AMC** | **TPZ** | **CZ** | **CRO** | **CXM** | **CPD** | **ETP** | **IMP** | **CN** | **CIP** | **SXT** | **CAZ CAZ-CV** | **CTX CTX-CV** | **ESBL** |
| --- | --- | --- | --- | --- | --- | --- | --- | --- | --- | --- | --- | --- | --- | --- | --- |
| 1 | R | R | R | R | R | R | R | S | S | R | R | R | R | R | Positive |
| 2 | R | S | S | R | S | S | S | S | S | S | S | S | S | S | Negative |
| 3 | R | R | R | R | R | R | R | S | S | S | R | R | R | R | Positive |
| 4 | R | R | R | R | R | R | R | R | S | R | R | R | R | R | Positive |
| 5 | R | R | R | R | R | R | R | S | S | R | R | R | R | R | Positive |
| 6 | R | S | S | R | S | S | S | S | S | S | S | S | S | S | Negative |
| 7 | R | R | R | R | R | R | R | S | S | R | R | R | R | R | Positive |
| 8 | R | R | R | R | S | S | S | S | S | S | S | S | S | S | Negative |
| 9 | R | S | S | R | S | S | S | S | S | S | S | S | S | S | Negative |
| 10 | R | R | R | R | R | R | R | S | S | S | R | R | R | R | Negative |
| 11 | R | S | S | R | S | S | S | S | S | S | S | S | S | S | Negative |
| 12 | R | S | S | R | S | S | S | S | S | S | S | R | S | S | Negative |
| 13 | R | S | S | R | S | S | S | S | S | S | S | R | S | S | Negative |
| 14 | R | R | R | R | R | R | R | R | S | R | R | R | R | R | Positive |
| 15 | R | S | S | R | S | S | S | S | S | S | S | S | S | S | Negative |
| 16 | R | R | R | R | R | R | R | S | S | S | R | R | S | S | Negative |
| 17 | R | R | S | R | S | S | S | S | S | S | S | R | S | S | Negative |
| 18 | R | S | S | R | S | S | S | S | S | S | S | S | S | S | Negative |
| 19 | R | R | R | R | R | R | R | S | S | S | R | R | S | R | Positive |
| 20 | R | R | S | R | S | S | S | S | S | R | R | S | S | S | Negative |
| 21 | R | R | R | R | R | R | R | S | S | R | R | R | R | R | Positive |
| 22 | R | S | S | R | S | S | S | S | S | S | S | S | S | S | Negative |
| 23 | R | R | R | R | R | R | R | R | R | S | R | R | R | R | Positive |
| 24 | R | R | R | R | R | R | R | S | S | S | R | R | R | R | Positive |
| 25 | R | S | S | R | S | S | S | R | S | S | R | R | R | R | Positive |
| 26 | R | R | R | R | R | R | R | R | R | S | R | R | R | R | Positive |
| 27 | R | R | S | R | S | S | S | S | S | R | S | R | S | S | Negative |
| 28 | R | S | S | R | S | S | S | S | S | S | S | S | S | S | Negative |
| 29 | R | S | S | R | S | S | S | S | S | S | S | S | S | S | Negative |
| 30 | R | R | R | R | R | R | R | S | S | S | R | R | R | R | Positive |
| 31 | R | S | R | S | S | S | S | S | S | S | S | S | S | S | Negative |
| 32 | R | R | R | R | R | R | R | S | S | R | R | R | R | R | Positive |
| 33 | R | R | R | R | R | R | R | R | S | S | S | R | R | R | Positive |
| 34 | R | R | R | R | R | R | R | S | S | R | R | R | R | R | Positive |
| 35 | R | R | R | R | R | R | R | R | R | S | S | S | S | R | Positive |
| 36 | R | R | R | R | R | R | R | S | S | S | R | R | R | R | Positive |
| 37 | R | S | S | R | S | S | S | S | S | S | S | S | S | S | Negative |
| 38 | R | R | R | R | R | R | R | R | R | S | R | R | R | R | Positive |
| 39 | R | S | S | S | S | S | S | S | S | S | S | S | S | S | Negative |
| 40 | R | S | S | R | S | S | S | S | S | S | S | S | S | S | Negative |
| 41 | R | R | R | R | R | R | R | S | S | S | S | R | R | R | Positive |
| 42 | R | R | R | R | R | R | R | R | R | S | R | R | R | R | Positive |
| 43 | R | S | S | R | S | S | S | S | S | S | S | R | S | S | Negative |
| 44 | R | S | S | R | S | S | S | S | S | S | S | S | S | S | Negative |
| 45 | R | R | S | R | S | S | S | S | S | S | S | R | S | S | Negative |
| 46 | R | R | R | R | R | R | R | S | S | R | R | R | R | S | Positive |
| 47 | R | R | R | R | S | R | S | S | S | R | R | R | S | S | Negative |
| 48 | R | R | R | R | R | R | R | R | S | R | R | R | R | R | Positive |
| 49 | R | R | R | R | R | R | R | S | S | S | S | R | R | R | Positive |
| 50 | R | R | R | R | R | R | R | S | S | S | R | R | R | R | Positive |
| 51 | R | R | R | R | R | R | R | R | S | S | R | R | R | R | Positive |
| 52 | R | R | S | R | S | S | S | S | S | S | S | S | S | S | Negative |
| 53 | R | R | R | R | R | R | R | R | S | R | R | R | R | R | Positive |
| 54 | R | S | S | S | S | S | S | S | S | S | S | R | S | S | Negative |
| 55 | R | R | S | R | S | S | S | S | S | R | R | S | S | S | Negative |
| 56 | R | S | S | R | S | S | S | S | S | S | S | R | S | R | Positive |
| 57 | R | S | S | R | S | S | S | S | S | S | S | S | S | S | Negative |
| 58 | R | S | S | R | S | S | S | S | S | S | S | S | S | S | Negative |
| 59 | R | S | S | R | S | S | S | S | S | S | R | R | S | S | Negative |
| 60 | R | R | R | R | R | R | R | R | S | R | R | R | R | R | Positive |
| 61 | R | R | R | R | R | R | R | R | R | S | R | R | R | R | Positive |
| 62 | R | S | S | R | S | S | S | S | S | S | S | S | S | S | Negative |
| 63 | R | S | S | R | S | S | S | S | R | S | S | S | S | S | Negative |
| 64 | R | S | S | R | S | S | S | S | R | S | S | S | S | S | Negative |
| 65 | R | S | S | R | S | S | S | S | S | S | S | S | S | S | Negative |
| 66 | R | R | S | R | R | R | R | S | S | R | S | S | R | R | Positive |
| 67 | R | R | R | R | R | R | R | S | S | R | R | R | R | R | Positive |
| 68 | R | R | R | R | R | R | R | S | S | S | R | R | S | S | Negative |
| 69 | R | R | R | R | R | R | R | S | S | R | R | R | R | R | Positive |
| 70 | R | S | S | R | R | S | S | S | S | S | S | S | S | S | Negative |
| 71 | R | R | R | R | S | S | S | S | S | R | R | R | S | S | Negative |
| 72 | R | S | S | S | S | S | S | S | S | S | S | S | S | S | Negative |
| 73 | R | S | S | R | S | S | S | S | S | S | S | S | S | S | Negative |
| 74 | R | S | S | R | S | S | S | S | S | S | S | S | S | S | Negative |
| 75 | R | R | R | R | R | R | R | S | S | S | R | R | S | S | Negative |
| 76 | R | S | S | S | S | S | S | S | S | S | S | S | S | S | Negative |
| 77 | S | S | S | S | S | S | S | S | S | S | S | S | S | S | Negative |
| 78 | R | R | R | R | R | R | R | R | R | S | R | R | R | R | Positive |
| 79 | R | S | S | R | S | S | S | S | S | S | S | S | S | S | Negative |
| 80 | R | R | R | R | R | R | R | R | R | S | S | S | S | R | Positive |
| 81 | R | S | S | S | S | R | R | S | S | S | R | R | S | S | Negative |
| 82 | R | S | S | R | R | R | R | S | S | S | R | R | R | R | Positive |
| 83 | R | S | S | R | R | R | R | S | S | R | R | R | R | R | Positive |
| 84 | R | R | R | R | R | R | R | S | S | R | S | R | R | R | Positive |
| 85 | R | R | R | R | R | R | R | S | S | S | R | R | R | R | Positive |
| 86 | R | S | S | R | S | S | S | S | S | S | S | S | S | S | Negative |
| 87 | R | S | S | R | S | S | S | R | S | S | S | S | S | S | Negative |
| 88 | R | S | S | R | S | S | S | S | S | S | S | S | S | S | Negative |
| 89 | R | R | R | R | R | R | R | R | S | S | S | R | S | S | Negative |
| 90 | R | R | R | R | R | R | R | S | S | S | S | R | R | R | Positive |
| 91 | S | S | S | R | S | S | S | S | S | S | S | S | S | S | Negative |
| 92 | R | R | R | R | R | R | R | S | S | S | S | S | R | R | Positive |
| 93 | R | R | S | R | R | R | R | S | S | S | R | R | R | R | Positive |
| 94 | R | S | S | S | S | S | S | S | S | S | S | S | S | S | Negative |
| 95 | R | R | R | R | R | R | R | R | R | R | R | R | R | R | Positive |
| 96 | R | R | R | R | S | S | S | S | S | S | S | S | S | S | Negative |
| 97 | R | R | R | R | R | R | R | R | S | S | S | R | R | S | Positive |
| 98 | R | S | S | S | S | S | S | S | S | R | S | S | S | S | Negative |
| 99 | R | R | R | R | R | R | R | R | R | R | R | R | S | S | Negative |

**Supplementary Data S4.** Antimicrobial agents used in ESBL screening and phenotyping.

| **Antimicrobial**  **Agent** | **Antimicrobial**  **Potency (µg)** | **Results** |
| --- | --- | --- |
| Ceftazidime | 30 | Inhibition zone ≤17mm may indicate  ESBL production |
| Ceftazidime-  clavulanic acid | 30/10 | A ≥ 5 mm increase in a zone diameter indicates  ESBL positive |
| Ceftriaxone | 30 | Inhibition zone ≤ 25mm may indicate  ESBL production |
| Ceftriaxone-  clavulanic acid | 30/10 | A ≥ 5 mm increase in a zone diameter  indicates ESBL positive |

**Supplementary Data S5.** Antimicrobial agents used in ESBL screening and phenotyping.

| **Antimicrobial**  **Agent** | **Antimicrobial**  **Potency (µg)** | **Results** |
| --- | --- | --- |
| Ceftazidime | 30 | Inhibition zone ≤17mm may indicate  ESBL production |
| Ceftazidime-  clavulanic acid | 30/10 | A ≥ 5 mm increase in a zone diameter indicates  ESBL positive |
| Ceftriaxone | 30 | Inhibition zone ≤ 25mm may indicate  ESBL production |
| Ceftriaxone-  clavulanic acid | 30/10 | A ≥ 5 mm increase in a zone diameter  indicates ESBL positive |

**Supplementary Data S6.** Antimicrobial agents used in Antimicrobial Susceptibility Testing.

| **Antimicrobial Agent** | **Antimicrobial Potency (µg**) |
| --- | --- |
|  |  |
| Ampicillin | 10 |
| Amoxicillin-clavulanic acid | 20/10 |
| Piperacillin-tazobactam | 100/10 |
| Cefazolin | 30 |
| Ceftriaxone | 30 |
| Cefuroxime | 30 |
| Cefpodoxime | 10 |
| Ertapenem | 10 |
| Imipenem | 10 |
| Gentamicin | 10 |
| Ciprofloxacin | 5 |
| Trimethoprim-sulfamethoxazole | 1.25/23.75 |

**Supplementary Data S7.** PCR results for isolates from hematological malignancy individuals.

| **Gene** | **Frequency of genes among ESBL-producers**  **(n=7)** | **Frequency of genes among non-ESBL producers (n=7)** |
| --- | --- | --- |
| *bla*_TEM_ | 4 (57.1%) | 3 (42.9%) |
| *bla*_SHV_ | 7 (100%) | 7 (100%) |
| *bla*_OXA_ | 2 (27.1%) | 1 (14.3%) |
| *bla*_CTX-M_ | 6 (85.7%) | 2 (28.6%) |

**Supplementary Data S8.** PCR results for isolates from individuals of various diagnosis.

| **Gene** | **Frequency of genes among ESBL-producers**  **(n=9)** | **Frequency of genes among non ESBL-producers**  **(n=8)** |
| --- | --- | --- |
| *bla*_TEM_ | 5 (55.5%) | 1 (12.5%) |
| *bla*_SHV_ | 9 (100%) | 6 (75%) |
| *bla*_OXA_ | 9 (100%) | 0 |
| *bla*_CTX-M_ | 9 (100%) | 0 |

**Supplementary Data S9.** List of primers used for PCR amplification.

| **Gene** | **Primer** | **Primer Sequence** | **Product size (bp)** | **Location** | **Reference** |
| --- | --- | --- | --- | --- | --- |
| *bla*_TEM_ | TEM-F TEM-R | 5´-ATGAGTATTCAACATTTCCG-3´  5´-CCAATGCTTAATCAGTGAGC-3´ | 857 | Plasmid | (27) |
| *bla*_SHV_ | SHV-F  SHV-R | 5´-CTTTACTCGCTTTATCG-3´  5´-TCCCGCAGATAAATCACCA-3´ | 800 | Plasmid | [27] |
| *bla*_OXA_ | OXA-1F  OXA-1R | 5´-ACACAATACATATCAACTTCGC-3´  5´-GTGTGTTTAGAATGGTGATC-3  ´ | 814 | Plasmid | [27] |
| *bla*_OXA_ | OXA-2 F  OXA- 2R | 5´-TTCAAGCCAAAGGCACGATAG-3´  5´-TCCGAGTTGACTGCCGGGTTG-3´ | 704 | Plasmid | [27] |
| *bla*_OXA_ | OXA-10 F  OXA-10 R | 5´-CGTGCTTTGTAAAAGTAGCAG-3´  5´-CATGATTTTGGTGGGAATGG-3´ | 651 | Plasmid | [27] |
| *bla*_CTX-M_ | CTX-MU1  CTX-MU2 | 5´-ATGTGCAGYACCAGTAARGT-3´  5´-TGGGTRAARTARGTSACCAGA-3´ | 593 | Plasmid | [27] |

**Supplementary Data S10**. Antibiotic resistance of *K. pneumoniae* isolates from hematological malignancy individuals.

| **Antimicrobial Agent** | **Susceptible %** | **Resistant (%)** |
| --- | --- | --- |
| Ampicillin  (Class: Penicillin) | 7.1 | 92.9 |
| Amoxicillin-clavulanic acid  (Class: Penicillin-Clavulanate) | 21.4 | 78.6 |
| Piperacillin-tazobactam  (Class: Penicillin) | 35.7 | 64.3 |
| Cefazolin  (Class: Cephalosporins) | 21.4 | 78.6 |
| Ceftriaxone  (Class: Cephalosporins) | 28.6 | 71.4 |
| Cefuroxime  (Class: Cephalosporins) | 28.6 | 71.4 |
| Cefpodoxime  (Class: Cephalosporins) | 28.6 | 71.4 |
| Ertapenem  (Class: Carbapenem) | 62.3 | 35.7 |
| Imipenem  (Class: Carbapenem) | 71.4 | 28.6 |
| Gentamicin  (Class: Aminoglycosides) | 50 | 50 |
| Ciprofloxacin  (Class: Fluoroquinolones) | 57.1 | 42.9 |
| Trimethoprim-sulfamethoxazole  (Sulfonamides) | 35.7 | 64.3 |

**Figure S3-A.** Electrophoresis of PCR products using primers for *bla*_TEM_ gene.

Lanes (1, 17): 100 bp ladder. Lane (2): Negative control. Lane (3): Positive control. Lane (4-10): ESBL-producing phenotype samples isolated from hematological malignancy patients, lanes (5, 6, 9, 10) are positive for *bla*_TEM_ gene. Four samples (57.1%) carried the *bla*_TEM_ gene. Lanes (11-17): non ESBL producing phenotype samples isolated from hematological malignancy patients, lanes (11, 12, 14): are positive for *bla*_TEM_ gene. Three samples (42.9%) carried the *bla*_TEM_ gene.


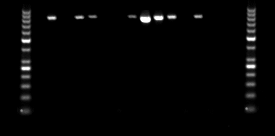


900bp

800bp

857 bp

1 2 3 4 5 6 7 8 9 10 11 12 13 14 15 17

**Figure S3-B.** Electrophoresis of PCR products using primers for *bla*_TEM_ gene.

Lanes (1): 100 bp ladder. Lanes (2-10): ESBL-producing phenotype samples isolated from non-hematological malignancy patients. Lanes (6-10) are positive for *bla*_TEM_ gene. Lane (11-17): non-ESBL producing phenotype samples isolated from non-hematological malignancy patients. Lane (12) is positive for *bla*_TEM_ gene.


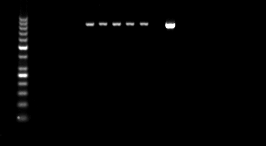


857 bp

800 bp

900 bp

1 2 3 4 5 6 7 8 9 10 11 12 13 14 15 16 17 18
